# Diversity of Oxacillinases and Sequence Types in Carbapenem-Resistant *Acinetobacter baumannii* from Austria

**DOI:** 10.3390/ijerph18042171

**Published:** 2021-02-23

**Authors:** Andrea J. Grisold, Josefa Luxner, Branka Bedenić, Magda Diab-Elschahawi, Michael Berktold, Agnes Wechsler-Fördös, Gernot E. Zarfel

**Affiliations:** 1 D&R Institute of Hygiene, Microbiology and Environmental Medicine, Medical University Graz, Neue Stiftingtalstrasse 6, A-8010 Graz, Austria; josefa.luxner@medunigraz.at (J.L.); gernot.zarfel@medunigraz.at (G.E.Z.); 2 Department of Microbiology, University Hospital Center Zagreb, 10000 Zagreb, Croatia; branka.bedenic@kbc-zagreb.hr; 3 Department of Infection Control and Hospital Epidemiology, Medical University of Vienna, Waehringer Guertel 18-20, 1090 Vienna, Austria; magda.diab-elschahawi@meduniwien.ac.at; 4 Institute of Hygiene and Microbiology, Medical University Innsbruck, Schöpfstrasse 41, A-6020 Innsbruck, Austria; michael.berktold@i-med.ac.at; 5 Hospital Rudolfstiftung, Juchgasse 25, 1030 Wien, Austria; foerdoes@aon.at

**Keywords:** *Acinetobacter baumannii*, *Acinetobacter pittii*, OXA carbapenemases, OXA-143, OXA-23, meropenem, imipenem

## Abstract

Carbapenem-resistant *Acinetobacter baumannii* is a significant health problem worldwide. A multicenter study on *A. baumannii* was performed to investigate the molecular epidemiology and genetic background of carbapenem resistance of *A. baumannii* isolates collected from 2014–2017 in Austria. In total, 117 non-repetitive *Acinetobacter* spp. assigned to *A. baumannii* (*n* = 114) and *A. pittii* (*n* = 3) were collected from four centers in Austria. The isolates were uniformly resistant to piperacillin/tazobactam, ceftazidime, and carbapenems, and resistance to imipenem and meropenem was 97.4% and 98.2%, respectively. The most prominent OXA-types were OXA-58-like (46.5%) and OXA-23-like (41.2%), followed by OXA-24-like (10.5%), with notable regional differences. Carbapenem-hydrolyzing class D carbapenemases (CHDLs) were the only carbapenemases found in *A.* *baumannii* isolates in Austria since no metallo-β-lactamases (MBLs) nor KPC or GES carbapenemases were detected in any of the isolates. One-third of the isolates harbored multiple CHDLs. The population structure of *A. baumannii* isolates from Austria was found to be very diverse, while a total of twenty-three different sequence types (STs) were identified. The most frequent was ST195 found in 15.8%, followed by ST218 and ST231 equally found in 11.4% of isolates. Two new ST types, ST2025 and ST2026, were detected. In one *A. pittii* isolate, *bla*_OXA-143-like_ was detected for the first time in Austria.

## 1. Introduction

The emergence of carbapenem resistance in *Acinetobacter baumannii* is a significant public health concern. Once *A. baumannii* was considered a low-category pathogen, and this organism now accounts for up to 20% of infections in ICUs [1,2], mainly associated with hospital acquired pneumonia and skin or soft tissue and bloodstream infections [3]. Along with *Enterococcus faecium*, *Staphylococcus aureus*, *Klebsiella pneumoniae*, *Pseudomonas aeruginosa*, and *Enterobacter* spp., *A. baumannii* is one of the ESKAPE pathogens of the WHO, which are on the priority list of antibiotic-resistant pathogens [4].

*A. baumannii* strains have emerged as resistant to almost all antimicrobial agents, including carbapenems, due to intrinsic or acquired resistance mechanisms [5]. The emergence of carbapenem-resistant, multidrug-resistant (MDR), extensively drug-resistant (XDR), or even pandrug-resistant *A. baumannii* has been progressively increasing over the last decade [6]. Carbapenem resistance mechanisms in *A. baumannii* are commonly mediated by carbapenem-hydrolyzing class D carbapenemases (CHDLs) [7], and less often by class B metallo-β-lactamases (MBLs) of the IMP, VIM, NDM, or SIM family [8,9,10] or, very rarely, class A carbapenemases (KPC, GES) [11]. However, *Acinetobacter* may develop resistance to carbapenems through various combined mechanisms, including decreased permeability, altering penicillin-binding proteins (PBPs) and, rarely, efflux pump overexpression [12]. OXA enzymes of *Acinetobacter* are divided into five phylogenetic groups: acquired enzymes OXA-23-like, OXA-24/40-like, OXA-58-like OXA-143-like, and OXA-235-like [5,13]. The enzymes of the OXA-51 group are chromosomally encoded in contrast to other previously mentioned groups which are usually plasmid-mediated and thus transferable. Enzymes belonging to the OXA-51 group are naturally occurring β-lactamases of *A. baumannii* and are normally expressed at low levels, but can be overexpressed as a consequence of the IS*Aba1* located upstream of the genes [7]. IS*Aba1* can be also located upstream of the other *bla*_OXA_genes and promote their expression [14].

There is a substantial difference in the incidence of *A. baumannii* infections between different countries. Particularly high rates of carbapenem resistance in *A. baumannii* in Europe are reported in Greece, Turkey, UK, Italy, and Croatia [15].

In Austria, there are only singular reports of local occurrence of carbapenem-resistant *A. baumannii* [16,17]. This prompted us to analyze the antibiotic resistance mechanisms and molecular epidemiology of *A. baumannii* isolates from different geographic regions in Austria.

## 2. Materials and Methods

### 2.1. Study Design and Bacterial Isolates

A total of presumptive 117 carbapenem-resistant *A. baumannii* isolates from different geographic regions in Austria were analyzed. Four microbiology laboratories provided isolates for analysis: Medical University of Graz, (D&R Institute of Hygiene, Microbiology and Environmental Medicine, Graz, Austria), Medical University of Innsbruck (Institute of Hygiene and Microbiology), Medical University Hospital Vienna (Department of Infection Control and Hospital Epidemiology), and Hospital Rudolfstiftung, Vienna. Geographically Graz is located in the province Styria in the south of Austria, Vienna located in the north, and Innsbruck is located in the province Tyrol, in the west of Austria. The *Acinetobacter* spp. isolates were collected from 2014 to 2017. All isolates were obtained from clinical samples, and only the first isolate per patient was included.

Microbiological and biochemical analysis was carried out at the Medical University of Graz (D&R Institute of Hygiene, Microbiology and Environmental Medicine). The isolates were kept at −80 °C before testing, then thawed and incubated onto blood agar for 24 h at 37 °C. The isolates were verified by MALDI-TOF (Vitek MS) and confirmed as *A. baumannii* by PCR for the *bla*_OXA-51_ gene in 114/117 isolates. Three isolates were identified as *Acinetobacter pittii* by MALDI-TOF (Vitek MS) and were analyzed separately. Sixty-nine *A. baumannii* isolates originated from Vienna, 33 from Innsbruck, and 12 from Graz. The three *A. pittii* isolates were all from Vienna.

The specimen originated in 87/117 (74.4%) from invasive samples (wound swab, bronchoalveolar lavage, blood cultures, urine, catheter tip, etc.) and 30/117 (25.6%) were from screening samples, like axilla, groin, forehead, or throat swab.

### 2.2. Antimicrobial Susceptibility Testing

The antimicrobial susceptibility testing for *A. baumannii* isolates was carried out using disc diffusion and E-test method. Included antimicrobial agents were imipenem, meropenem, piperacillin/tazobactam, ampicillin/sulbactam, cefepime, ceftazidime, ciprofloxacin, gentamicin, amikacin, trimethoprim/sulfamethoxazole, colistin, and tigecycline. *Pseudomonas aeruginosa* ATCC 27853 and *Acinetobacter baumannii* 19606 were used as quality control strains. Results were interpreted according to the Clinical and Laboratory Standards Institute (CLSI) recommendations [18]. Modified Hodge test was performed as described previously [19]. Isolates were defined as multidrug-resistant (MDR), extensively drug-resistant (XDR) or pandrug-resistant (PDR) according to Magiorakos et al. [20].

### 2.3. Molecular Identification of β-Lactamases

The presence of the genes encoding the MBLs was investigated by PCR. DNA was isolated by suspending one colony of an isolate in 50 µL ultra-pure water. Suspensions were heated at 95 °C for 10 min and, afterwards, samples were chilled on ice for one minute and centrifuged in Eppendorf TM Centrifuge 5415 at maximum speed for 1 min. Supernatant was used as template for PCR. The genes *bla*_OXA-23-like_, *bla*_OXA-51-like,_ *bla*_OXA-24-like_, *bla*_OXA-58-like_, *bla*_OXA-143-like_, *bla*_OXA-235-like_, *bla*_IMP,_ *bla*_SIM_ *bla*_VIM_, *bla*_KPC_, *bla*_GES_, and *bla*_TEM_ were amplified using Gene Am PCR System 9700 (Applied Biosystems) using previously described primers [21,22,23,24,25,26], and are also listed in Supplementary Table S1. The amplification conditions were as follows: initial denaturation at 95 °C for 5 min; 35 cycles of 94 °C for 30 s, 52 °C for 45 s, and 72 °C for 60 s; and a final elongation at 72 °C for 10 min.

Strains with sequenced, confirmed resistance genes were used as controls. Positive control strains harboring bla_OXA-23_, bla_OXA-24_, bla_OXA-58_, and bla_OXA-235_ genes were kindly provided by Dr. Paul Higgins, Institute for Microbiology and Hygiene, University of Cologne, Cologne, Germany. In addition, the *bla*_TEM_ genes were also searched for (as a marker for the uptake of non-OXA β lactamases in *A. baumannii*). The positive control strain producing TEM-1 β-lactamase was kindly obtained from Prof. Adolf Bauernfeind, Max von Pettenkofer Institute, Munich, Germany.

### 2.4. Multi-Locus Sequence Analysis

MLST was performed according to Diancourt et al. [27], and sequence type (ST) results were retrieved from the database available from Acinetobacter MLST (Oxford) databases (https://pubmlst.org/abaumannii/ (accessed on 13 June 2019).

## 3. Results

### 3.1. Antimicrobial Susceptibility Testing

From the total of 117 presumptive *A. baumannii* 114 isolates were confirmed as *A. baumannii* and susceptibility is described here in detail. A total of 101/114 (88.6%) isolates were multidrug-resistant (MDR), 12 (10.5%) were extensively-drug-resistant (XDR), and one isolate (0.9%) was pan-resistant (PDR). The isolates were uniformly resistant to piperacillin/tazobactam, cefepime, and ceftazidime. Resistance to imipenem and meropenem was 97.4% (111/114) and 98.2% (112/114), respectively. The majority of isolates were resistant to ciprofloxacin (98.2%, 112/114), gentamicin (88.6%, 101/114), amikacin and trimethoprim/sulfamethoxazole (92.1%, each 105/114), and ampicillin/sulbactam (95/114, 83.3%). High susceptibility rates were observed for colistin (94.7%, 108/114) and tigecycline (93.0%, 106/114). Modified Hodge test was positive in 82.5% (94/114) isolates indicating carbapenemase activity. Susceptibility of the three *A. pittii* isolates is depicted in detail at the end of Supplementary Table S1.

### 3.2. Detection of β-lactamases Genes

**Figure 1:**
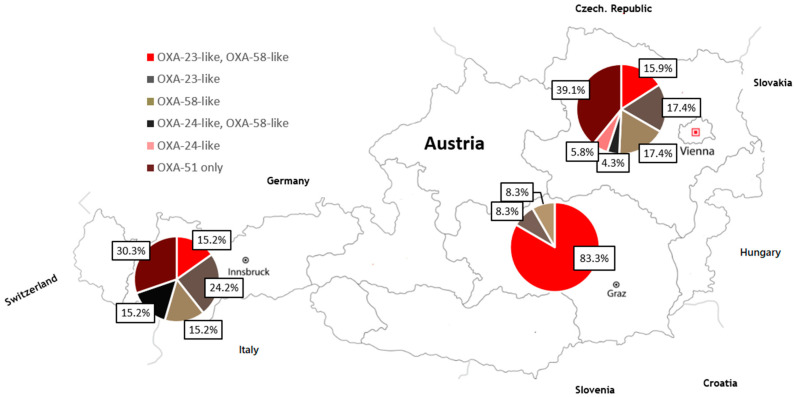
Geographical distribution of carbapenemase-encoding OXA-type genes in carbapenem-resistant *A. baumannii* isolates in Austria.

All *A. baumannii* isolates were, as expected, positive for the intrinsic *bla*_OXA-51_ gene and were subjected to molecular detection of genes encoding acquired β-lactamases. Fifty-three out of 114 isolates (46.5%) harbored genes encoding OXA-58-like CHDL. Genes for OXA-23-like was found in 47 isolates (41.2%) (Supplementary Table S1). *bla*_OXA-24-like_ was identified in only 12 isolates (10.5%), whereas none of the *A. baumannii* isolates in Styria, in the south of Austria, harbored *bla*_OXA-24-like_. The simultaneous existence of genes encoding for OXA-23-like and OXA-58-like CHDL was identified in 26 isolates (22.8%). Co-existence of genes encoding for OXA-24-like and OXA-58-like CHDL was found in eight isolates (7.0%), whereas five of those occurred in the West of Austria(2017). Twenty-eight *A. baumannii* isolates (24.6%) harbored *bla*_OXA-51_ only, with 8 isolates in Innsbruck (2014 and 2016) and 20 isolates in Vienna (throughout study period). None of OXA-51 only isolates were identified in Styria. The regional differences are depicted in detail in Figure 1. Thirty-seven isolates (32.4%) tested positive for *bla*_TEM_ genes, whereas *bla*_TEM_ was most prevalent in Vienna (69%, 30/69 isolates), and none of the isolates of Styria harbored TEM β-lactamases. It is noteworthy that in none of the isolates were genes encoding MBLs and KPC or GES carbapenemases found (Supplementary Table S1).

In one of the three *A. pittii* isolates, the only detected CHDL was OXA-143-like, whereas the second one harbored genes encoding for OXA-23-like and TEM. One *A. pittii* isolate was negative for all tested genes, including *bla*_OXA-51_ (Supplementary Table S1).

### 3.3. Genotyping/MLST

**Figure 2:**
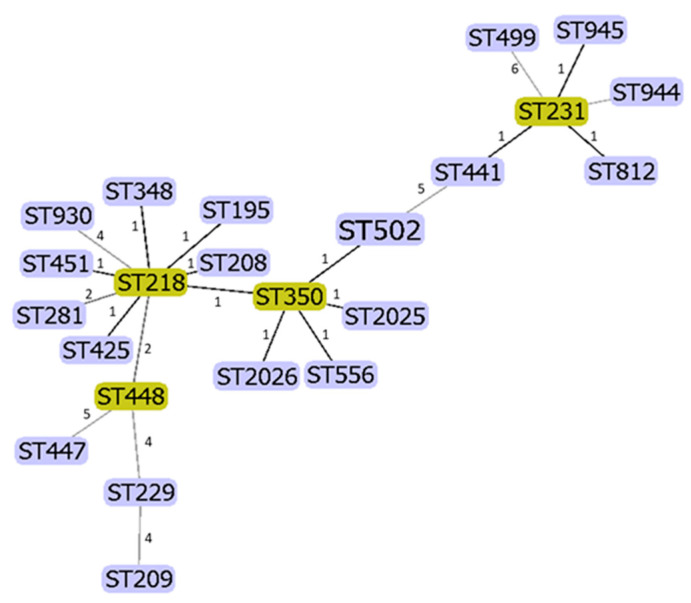
Dendrogram of detected MLSTs in *A. baumannii* in Austria.

The population structure and sequence types (ST) of *A. baumannii* isolates from Austria was very diverse. Within the 114 isolates, twenty-three different STs were found (Figure 2). The most frequent was ST195, found in 18 (15.8%) of the isolates. ST218 and ST231 were equally found in thirteen isolates (11.4%), followed by ST208 (11 isolates, 9.6%), ST425, ST451, and ST502 (eight isolates each, 7.1%), ST348 (seven isolates, 6.1%), ST350 (six isolates, 5.3%), and ST281 (four isolates, 3.5%) (Table 1). All other ST types were found with only one or two isolates (Table 1).

Dendrogram was calculated using goeBURST full MST algorithm using PHYLOViZ software (https://phyloviz.readthedocs.io/en/latest/index.html (accessed on 16 February 2021). Numbers on the lines indicate the distance between the types.

Allocating to the different regions in Austria, the most dominant ST in Styria/south of Austria was ST218, followed by ST195 (three isolates/25%), in Tyrol/west of Austria the majority of the isolates were assigned to ST451 and ST348, followed by ST218, ST231, and ST281. In Vienna in the north of Austria, the most prevalent ST was ST195, followed by ST208, ST231, and ST425.

Three MLST types, ST195, ST218, and ST231 were found at all three geographical locations. The ST195 isolates found in Styria carried the blaOXA-23-like and blaOXA-58-like, whereas the two ST195 isolates from Tyrol carried blaOXA-23-like gene only. The thirteen ST195 isolates from Vienna carried blaOXA-23-like alone (six isolates) or blaOXA-23-like and blaOXA-58-like (four isolates), or blaOXA-58-like alone (two isolates), whereas one isolate harbored intrinsic blaOXA-51 only. ST218 also looks rather diverse. The ST218 isolates from Styria were found in 2014 only and were homogeneously positive for *bla*_OXA-23-like_ and *bla*_OXA-58-like_. The ST218 isolates from Tyrol were found in 2016 and 2017 only, and harbored *bla*_OXA-58-like_ only. Only two ST218 isolates were found in Vienna, both in 2016, and did not possess any acquired CHDLs. ST231 was found in 2014 (two isolates) and 2016 (one isolate) in Innsbruck, a single isolate was found 2016 in Styria and nine isolates occurred in Vienna, three in 2014, four in 2016 and two in 2017 (Supplementary Table S1).

Two new Oxford *A. baumannii* MLST types were found in the investigated *A. baumannii* isolates in Austria. ST2025 in one isolate from Vienna and ST2026 in one isolate from Vienna and one from Styria, respectively. Interestingly, although both ST2026 isolates were found in 2017, the *bla*_OXA_ genes content differed. The isolate from Vienna contained the bla_OXA-23-like_ gene as an additional *bla*_OXA_ gene, while the Styrian isolate contained *bla*_OXA-58-like_ only.

## 4. Discussion

Carbapenem-resistant *A. baumannii* has emerged as a major culprit involved in nosocomial infections, especially in intensive care units [1,2,28].

Naturally, this organism occurs in many different habitats, including food, soil, or surface water [29,30,31]. Recently concerns have also been raised about the possibility of transmission of multiresistant *A. baumannii* between animals and humans. The occurrence of this pathogen in livestock represents a further notable risk of transmission to humans and further into hospitals [32,33]. The capability of *A. baumannii* to survive harsh conditions, including disinfection, fosters its persistence in hospital environments.

Numerous nosocomial outbreaks involving *A. baumannii* have been reported and especially carbapenem-resistant *A. baumannii* is now endemic in many hospitals worldwide [28,34]. Several resistance mechanisms against carbapenems in *A. baumannii* have been reported, and for many countries, the molecular epidemiology of carbapenem and/or MDR *A. baumannii* is known [28].

For Austria, this is the first mapping of involved carbapenem resistance mechanisms in *A. baumannii*. The main findings of this study are that throughout the entire study period, CHDLs were the only carbapenemases found among the isolates. Despite the large number of isolates, no MBLs nor KPC or GES carbapenemases were found.

Within the isolates, a high rate of co-existence of different CHDLs was observed, and there was also a pronounced diversity of the found STs, with a simultaneous existence of *bla*_oxa23-like_ and *bla*_oxa58-like_ in 22.8% and co-existence of *bla*_oxa24-like_ and *bla*_oxa58-like_ in 7.0%.

OXA-23 was the first carbapenem-hydrolyzing oxacillinase reported in 1986 in UK as ARI-1 [35]. It was later renamed to OXA-23. It has spread all over the world, but most reports originate from Europe [36] and the Far East [37,38], but also from South America [39]. It is the dominant type of CHDL in Bulgaria [40], Germany [41], and Italy [42]. OXA-23 was recently reported in the Austria’s neighboring country Croatia in a multicenter study conducted in 2009–2010 [43], in Istria [44], and it was also previously found in a nursing home in Zagreb [45].

OXA-58 was the most frequent type of CHDL reported in this study. It is the most frequent CHDL in Turkey, France [14,46,47], and Greece [48,49]. OXA-24-like is the most prevalent group in Balkan countries, with OXA-72 as only allelic variant reported, often associated with nosocomial outbreaks [50,51,52,53]. In Austria, genes encoding OXA-24-like were found in twelve *A. baumannii* isolates with appreciable regional differences. OXA-24-like was not found in isolates from Styria in the south, and *bla*_TEM_ was also not found in those isolates. Of note, a few strains were found to possess intrinsic *bla*_OXA-51_ only.

Almost one-third of the isolates harbored multiple CHDLs which was rare in previous studies pointing out to the increasing capacity of *A. baumannii* to accumulate carbapenem resistance determinants [28].

The *A. baumannii* isolates with the same type of CHDL in this study belonged to different STs, indicating the lack of correlation between the population structure and acquired oxacillinases. Take the MLST typing and gene data into account, only one outbreak with six isolates (South Austria, 2014) took place. Other nosocomial events could not be excluded but were, in any case, rare and limited to less than five patients, which is in accordance with previous reports from Austria [16,17].

Although *A. baumannii* is the most clinically relevant *Acinetobacter* species, there are increasing reports of infections caused by non-*baumannii Acinetobacter* species. Within this study, three *Acinetobacter* spp. isolates, primarily identified as *A. baumannii* were identified as *A. pittii* when retested. One of these isolates harbored a gene encoding for OXA-143, a rare type of CHDL previously found in Iraq, Brazil, USA, and Peru [54,55,56].

Antibiotic therapy for carbapenem-resistant *A. baumannii* is challenging. With the increase in the use of colistin to treat carbapenem-resistant *A. baumannii* infections, colistin resistance is emerging worldwide [57,58,59]. This study revealed that resistance to colistin is still rare in Austria [16,60], and the same was noted for tigecycline. High resistance rates were observed for ciprofloxacin, gentamicin, and amikacin.

Regarding the population structure, the *A. baumannii* isolates from Austria were found to be very diverse, with a total of 23 different sequence types (STs).

ST195, which is dominant in Austria, is also the most frequent ST in Croatia, a country with a lot of students and personal exchange with Austria. Notably, *A. baumannii* isolates with ST195 have also recently been detected in two isolates in a Croatian pig farm [32], from Austria, as a country with intensive animal husbandry, up to now, no cases have yet been reported. OXA-72 is the dominant CHDL in all geographic regions of Croatia in contrast to Austria in which OXA-58 predominates [42,50].

The high diversity of STs was also observed between different geographic regions of Austria in spite of the fact that the same CHDLs were found everywhere in Austria. In addition, two new ST types were found, ST2025 and ST2026.

## 5. Conclusions

Carbapenem-resistant *A. baumannii* has become a global problem in healthcare settings. This is the first study on the epidemiology and molecular characterization of *A. baumannii* from a hospital setting in Austria and closes a gap in the mapping of the worldwide distribution of this pathogen.

The main findings were that CHDLs were the only carbapenemases found in *A. baumannii* isolates in Austria since MBLs and KPC or GES carbapenemases as an underlying resistance mechanism were not detected in any of the isolates. OXA-23-like and OXA-58-like were the most prominent OXA-types, followed by OXA-24-like, but with notable regional differences. Interestingly one third of the isolates harbored multiple CHDLs.

The population structure the *A. baumannii* isolates from Austria was found to be very diverse, with a total of 23 different sequence types (STs).

The results demonstrate that there is no endemic *A. baumannii* strain circulating in Austria, indicating that there is a frequent import of isolates via incomers, tourists, students, or patients after a stay in a hospital abroad emphasizing, once more, the necessity of hospital hygiene measurements like screening to prevent nosocomial outbreaks. In addition, the environmental reservoirs may contribute to this diversity.

Moreover, this is the first report of OXA-143 in Europe and the first report in *A. pittii*, demonstrating the ability of *bla*_OXA_ genes to spread between different species in the genus *Acinetobacter* spp.

## Figures and Tables

**Table 1:** Multilocus sequence typing (MLST) and sequence types (ST) of carbapenem-resistant *A. baumannii* isolates in Austria.

REGION/AUSTRIA		SEQUENCE TYPE														
	195	218	231	208	425	451	502	348	350	281	2026	441	448	812	945	Others ^1^
South (*n* = 12)	3	6	1	-	-	-	-	-	-	-	1	-	-	-	-	1
West (*n* = 33)	2	5	3	1	1	7	1	7	1	3	-	-	-	-	-	2
North (*n* = 69)	13	2	9	10	7	1	7	-	5	1	1	2	2	2	2	5
Total (*n* = 114/%)	18 15.8%	13 11.4%	13 11.4%	11 9.7%	8 7.0%	8 7.0%	8 7.0%	7 6.1%	6 5.3%	4 3.5%	2 1.8%	2 1.8%	2 1.8%	2 1.8%	2 1.8%	8 7.0%

^1^ Others: ST202, ST209, ST229, ST447, ST499, ST556, ST944, ST930, and 2025 were found in one isolate per year or region only.

## Data Availability

The data presented in this study are available in [article].

## Supplementary Materials

The following are available online at https://www.mdpi.com/1660-4601/18/4/2171/s1, Table S1: Target and Primers used in this study and respective literature; Table S2: Antibiotic susceptibility, genotypes, and β-lactamase content of *A. baumannii* in Austria.
