# Supplementary material for: Diversity of Oxacillinases and Sequence Types in Carbapenem-Resistant Acinetobacter baumannii from Austria
Source: Int J Environ Res Public Health. 2021 Feb 23;18(4):2171. doi: 10.3390/ijerph18042171 (PMC7926329; doi:10.3390/ijerph18042171)
Supplement: Supplementary file 1 [file ijerph-18-02171-s001.pdf]

International Journal of
Environmental Research and Public Health

Supplementary Table S1. Target and Primers used in this study and respective literature.

Primer Name	Sequence (5´->3´)	Target	Reference
OXA-23-likeF	ACAGAARTATTTAAGTGGG	*bla*$_{OXA-23-like}$	Woodford et al. [21]
OXA-23-likeR	ATT TCT GAC CGC ATT TCC AT		
OXA-24-likeF	GGT TAG TTG GCC CCC TTA AA	*bla*$_{OXA-24-like}$	Woodford et al. [21]
OXA-24-likeR	AGT TGA GCG AAA AGG GGA TT		
OXA-51-F	TAA TGC TTT GAT CGG CCT TG	*bla*$_{OXA-51}$	Woodford et al. [21]
OXA-51-R	TGG ATT GCA CTT CAT CTT GG		
OXA-58-likeF	AAG TAT TGG GGC TTG TGC TG	*bla*$_{OXA-58-like}$	Woodford et al. [21]
OXA-58-likeR	CCC CTC TGC GCT CTA CAT AC		
VIM-F	GAT GGT GTT TGG TCG CAT A	*bla*$_{VIM}$	Poirel et al. [22]
VIM-R	CGA ATG CGC AGC ACC AG		
IMP-F	GGA ATA GAG TGG CTT AAY TCT C	*bla*$_{IMP}$	Poirel et al. [22]
IMP-R	GGT TTA AYA AAA CAA CCA CC		
SIM-F	AAA ATC TGG GTA CGC AAA CG	*bla*$_{SIM}$	Poirel et al. [22]
SIM-R	ACA TTAT CCG CTG GAA CAG G		
NDM-F	GGT TTG GCG ATC TGG TTT TC	*bla*$_{NDM}$	Poirel et al. [22]
NDM-R	CGG AAT GGC TCA TCA CGA TC		
KPC	CGT CTA GTT CTG CTG TCT TG	*bla*$_{KPC}$	Poirel et al. [22]
KPC	CTT GTC ATC CTT GTT AGG CG		
OXA-143-F	TGG CAC TTT CAG CAG TTC CT	*bla*$_{OXA-143-like}$	Higgins et al. [23]
OXA-143-R	TAA TCT TGA GGG GGC CAA CC		
OXA-235-F	CAA GCC ATG CAA GCT TCT	*bla*$_{OXA-235-like}$	Higgins et al. [24]
OXA-235-R	GCT GGA CTT GAG GAT CAA AG		
TEM-F	ATG AGT ATT CAA CAT TTC CG	*bla*$_{TEM}$	Eckert et al 2004 [25]
TEM-R	CCA ATG CTT AAT CAG TGA GG		
GES-F	AGTCGGCTAGACCGGAAAG	*bla*$_{GES}$	Dallenne et. al [26]
GES-R	TTTGTCCGTGCTCAGGAT		

Supplementary Table S2. Antibiotic susceptibility, genotypes, and β-lactamase content of *A. baumannii* in Austria.

No.	Year	Region	Specimen	MDR /XDR /PDR	Resistance Pattern													ß-lactamase content	Sequence Type
					SAM	TZP	CAZ	FEP	IMP	MEM	GM	AN	SXT	CIP	CL	TGC	Hodge		
1	2014	Styria/ South Austria	Urine	MDR	R	R	R	R	R	R	R	R	R	R	S	S	pos	OXA-23-like, OXA-58-like, OXA-51	ST218
2	2014	Styria/ South Austria	Tracheal aspirate	MDR	R	R	R	R	R	R	R	R	R	R	S	S	pos	OXA-23-like, OXA-58-like, OXA-51	ST218
3	2014	Styria/ South Austria	Wound swab	MDR	R	R	R	R	R	R	R	R	R	R	S	S	pos	OXA-23-like, OXA-58-like, OXA-51	ST218
4	2014	Styria/ South Austria	Throat swab	MDR	R	R	R	R	R	R	R	R	S	R	S	S	pos	OXA-23-like, OXA-58-like, OXA-51	ST218
5	2014	Styria/ South Austria	Groin	MDR	R	R	R	R	R	R	R	R	R	R	S	S	pos	OXA-23-like, OXA-58-like, OXA-51	ST218
6	2014	Styria/ South Austria	Wound swab	MDR	R	R	R	R	R	R	R	R	R	R	S	S	pos	OXA-23-like, OXA-58-like, OXA-51	ST218
7	2015	Styria/ South Austria	Tracheal aspirate	MDR	R	R	R	R	R	R	R	R	R	R	S	S	pos	OXA-23-like, OXA-58-like, OXA-51	ST195
8	2016	Styria/ South Austria	Urine	MDR	R	R	R	R	R	R	R	R	S	R	S	S	neg	OXA-23-like, OXA-58-like, OXA-51	ST195
9	2016	Styria/ South Austria	Stool	MDR	R	R	R	R	R	R	R	R	S	R	S	S	pos	OXA-23-like, OXA-58-like, OXA-51	ST195
10	2016	Styria/ South Austria	Wound swab	MDR	R	R	R	R	R	R	R	R	R	R	S	S	pos	OXA-23-like, OXA-51-like	ST231
11	2017	Styria/ South Austria	Urine	MDR	R	R	R	R	R	R	R	R	R	R	S	S	pos	OXA-23-like, OXA-58-like, OXA-51	ST229

12	2017	Styria/ South Austria	Wound swab	MDR	R	R	R	R	R	R	R	R	R	R	S	S	neg	OXA-58-like, OXA-51	ST2026
13	2014	Innsbruck/ West Austria	Wound swab	MDR	R	R	R	R	R	R	R	R	R	R	S	S	pos	OXA-51	ST348
14	2014	Innsbruck/ West Austria	Groin	MDR	R	R	R	R	R	R	R	R	R	R	S	S	pos	OXA-51	ST348
15	2014	Innsbruck/ West Austria	Sputum	MDR	R	R	R	R	R	R	R	R	R	R	S	S	pos	OXA-51	ST944
16	2014	Innsbruck/ West Austria	Blood culture	MDR	R	R	R	R	R	R	R	R	R	R	S	S	pos	OXA-51	ST502
17	2014	Innsbruck/ West Austria	Axilla	MDR	R	R	R	R	R	R	R	R	R	R	S	S	neg	OXA-23-like, OXA-51	ST281
18	2014	Innsbruck/ West Austria	Urine	MDR	R	R	R	R	R	R	R	R	R	R	S	S	pos	OXA-23-like, OXA-51, TEM	ST231
19	2014	Innsbruck/ West Austria	Groin	MDR	R	R	R	R	R	R	R	R	R	R	S	S	pos	OXA-23-like, OXA-51, TEM	ST231
20	2015	Innsbruck/ West Austria	Wound swab	MDR	R	R	R	R	R	R	R	S	R	R	S	S	neg	OXA-23-like, OXA-51	ST195
21	2015	Innsbruck/ West Austria	Drain	MDR	R	R	R	R	R	R	R	R	S	R	S	S	pos	OXA-23-like, OXA-51, TEM	ST208
22	2015	Innsbruck/ West Austria	Urine	MDR	R	R	R	R	R	R	R	R	R	R	S	S	pos	OXA-23-like, OXA-51	ST451
23	2015	Innsbruck/ West Austria	Decubital ulcer	MDR	R	R	R	R	R	R	R	R	R	R	S	S	pos	OXA-23-like, OXA-51, TEM	ST451
24	2015	Innsbruck/ West Austria	Urine	MDR	R	R	R	R	R	R	R	R	R	R	S	S	pos	OXA-23-like, OXA-51	ST195

25	2015	Innsbruck/ West Austria	Urine	MDR	R	R	R	R	R	R	R	R	R	R	S	S	pos	OXA-58-like, OXA-51	ST451
26	2015	Innsbruck/ West Austria	Forehead	MDR	R	R	R	R	R	R	R	R	R	R	S	S	pos	OXA-23-like, OXA-58-like, OXA-51	ST350
27	2015	Innsbruck/ West Austria	Wound drain	MDR	R	R	R	R	R	R	S	R	R	R	S	S	pos	OXA-23-like, OXA-58-like, OXA-51	ST425
28	2016	Innsbruck/ West Austria	Throat swab	MDR	R	R	R	R	R	R	R	R	R	R	S	S	pos	OXA-51	ST281
29	2016	Innsbruck/ West Austria	Tracheal aspirate	MDR	R	R	R	R	R	R	R	R	R	R	S	S	pos	OXA-51, TEM	ST451
30	2016	Innsbruck/ West Austria	Groin	MDR	R	R	R	R	R	R	S	R	R	R	S	S	pos	OXA-58-like, OXA-51	ST231
31	2016	Innsbruck/ West Austria	Groin	MDR	R	R	R	R	R	R	R	R	R	R	S	S	pos	OXA-51	ST451
32	2016	Innsbruck/ West Austria	Tracheal aspirate	MDR	R	R	R	R	R	R	R	R	R	R	S	S	pos	OXA-58-like, OXA-51	ST281
33	2016	Innsbruck/ West Austria	Tracheal aspirate	MDR	R	R	R	R	R	R	R	R	R	R	S	S	pos	OXA-58-like, OXA-51, TEM	ST451
34	2016	Innsbruck/ West Austria	Sputum	MDR	R	R	R	R	R	R	R	R	R	R	S	S	pos	OXA-58-like, OXA-51	ST218
35	2016	Innsbruck/ West Austria	Wound swab	MDR	R	R	R	R	R	R	R	S	R	R	S	S	pos	OXA-51	ST451
36	2016	Innsbruck/ West Austria	Tracheal aspirate	MDR	R	R	R	R	R	R	S	S	R	R	S	S	pos	OXA-51	ST218
37	2016	Innsbruck/ West Austria	Urine	MDR	R	R	R	R	R	R	S	S	R	S	S	S	pos	OXA-51, TEM	ST556

38	2017	Innsbruck/ West Austria	Nose	MDR	S	R	R	R	R	R	R	R	R	R	S	S	pos	OXA-24-like, OXA-58-like, OXA-51	ST348
39	2017	Innsbruck/ West Austria	Sputum	MDR	S	R	R	R	R	R	R	R	R	R	S	S	neg	OXA-23-like, OXA-58-like, OXA-51	ST218
40	2017	Innsbruck/ West Austria	Nose	MDR	S	R	R	R	R	R	R	R	R	R	S	S	pos	OXA-24-like, OXA-58-like, OXA-51	ST348
41	2017	Innsbruck/ West Austria	Groin	MDR	S	R	R	R	R	R	R	S	R	R	S	S	pos	OXA-23-like, OXA-58-like, OXA-51	ST218
42	2017	Innsbruck/ West Austria	Tracheal aspirate	MDR	S	R	R	R	R	R	R	R	R	R	S	S	pos	OXA-24-like, OXA-58-like, OXA-51	ST348
43	2017	Innsbruck/ West Austria	Tracheal aspirate	MDR	S	R	R	R	R	R	R	R	R	R	S	S	pos	OXA-24-like, OXA-58-like, OXA-51	ST348
44	2017	Innsbruck/ West Austria	Bronchoalveolar lavage	MDR	R	R	R	R	R	R	R	R	R	R	S	S	pos	OXA-23-like, OXA-58-like, OXA-51, TEM	ST218
45	2017	Innsbruck/ West Austria	Decubital ulcer	MDR	S	R	R	R	R	R	S	R	R	R	S	S	pos	OXA-24-like, OXA-58-like, OXA-51	ST348
46	2014	Vienna/ North Austria	Stool	MDR	R	R	R	R	R	R	R	R	R	R	S	S	pos	OXA-23-like, OXA-51	ST441
47	2014	Vienna/ North Austria	Wound swab	XDR	R	R	R	R	R	R	R	R	R	R	R	S	pos	OXA-24-like, OXA-51	ST441
48	2014	Vienna/ North Austria	Tracheal aspirate	PDR	R	R	R	R	R	R	R	R	R	R	R	R	neg	OXA-23-like, OXA-51, TEM	ST425
49	2014	Vienna/ North Austria	Bloodculture	XDR	I	R	R	R	R	R	R	R	R	R	S	R	pos	OXA-51	ST231
50	2014	Vienna/ North Austria	Bronchoalveolar lavage	MDR	R	R	R	R	R	R	R	R	R	R	S	S	pos	OXA-58-like, OXA-51, TEM	ST208

51	2014	Vienna/ North Austria	Wound swab	MDR	S	R	R	R	R	R	R	R	R	R	S	S	pos	OXA-24-like, OXA-58like, OXA-51	ST502
52	2014	Vienna/ North Austria	Stool	MDR	R	R	R	R	R	R	R	R	R	R	S	S	pos	OXA-24-like, OXA-58-like#, OXA-51	ST350
53	2014	Vienna/ North Austria	Catheter tip	MDR	I	R	R	R	R	R	R	R	S	R	S	S	pos	OXA-24-like, OXA-58-like, OXA-51	ST2025
54	2014	Vienna/ North Austria	Bronchoalveolar lavage	MDR	S	R	R	R	R	R	R	R	R	R	S	S	pos	OXA-24-like, OXA-51	ST231
55	2014	Vienna/ North Austria	Bronchoalveolar lavage	MDR	R	R	R	R	R	R	R	R	R	R	S	S	pos	OXA-58-like, OXA-51, TEM	ST448
56	2014	Vienna/ North Austria	Tracheal aspirate	MDR	R	R	R	R	R	R	S	R	R	R	S	S	pos	OXA-24-like, OXA-51	ST231
57	2014	Vienna/ North Austria	Sputum	MDR	R	R	R	R	R	R	R	R	R	R	S	S	pos	OXA-58-like, OXA-51, TEM	ST448
58	2014	Vienna/ North Austria	Bronchoalveolar lavage	MDR	R	R	R	R	R	R	S	R	R	R	S	S	pos	OXA-58-like, OXA-51	ST499
59	2014	Vienna/ North Austria	Catheter tip	MDR	R	R	R	R	R	R	R	R	R	R	S	S	pos	OXA-51	ST451
60	2014	Vienna/ North Austria	Wound swab	MDR	R	R	R	R	R	R	R	R	R	R	S	S	pos	OXA-58-like, OXA-51	ST350
61	2014	Vienna/ North Austria	Tracheal aspirate	MDR	R	R	R	R	R	R	R	R	R	R	S	S	pos	OXA-23-like, OXA-58-like, OXA-51, TEM	ST195
62	2015	Vienna/ North Austria	Tracheal aspirate	MDR	R	R	R	R	R	R	R	R	R	R	S	S	pos	OXA-23-like, OXA-51, TEM	ST208
63	2015	Vienna/ North Austria	Stool	XDR	R	R	R	R	R	R	R	R	R	R	R	S	neg	OXA-23-like, OXA-51, TEM	ST208

64	2015	Vienna/ North Austria	Central venous catheter tip	XDR	R	R	R	R	R	R	R	R	R	R	S	R	pos	OXA-23-like, OXA-51	ST195
65	2015	Vienna/ North Austria	Bronchoalveolar lavage	MDR	R	R	R	R	R	R	S	R	R	R	S	S	pos	OXA-23-like, OXA-58-like, OXA-51	ST350
66	2015	Vienna/ North Austria	Nose	MDR	R	R	R	R	R	R	R	R	R	R	S	S	pos	OXA-58-like, OXA-51	ST350
67	2015	Vienna/ North Austria	Sputum	MDR	S	R	R	R	R	R	R	R	R	R	S	S	neg	OXA-23-like, OXA-51, TEM	ST945
68	2015	Vienna/ North Austria	Wound swab	MDR	S	R	R	R	R	R	R	R	R	R	S	S	pos	OXA-23-like, OXA-58	ST945
69	2015	Vienna/ North Austria	Throat swab	MDR	R	R	R	R	R	R	R	R	R	R	S	S	pos	OXA-23-like, OXA-58-like, OXA-51, TEM	ST208
70	2015	Vienna/ North Austria	Stool	MDR	R	R	R	R	R	R	R	R	R	6	S	S	pos	OXA-23-like, OXA-58-like, OXA-51, TEM	ST208
71	2015	Vienna/ North Austria	Rectal swab	MDR	R	R	R	R	S	R	R	R	R	R	S	S	pos	OXA-58-like, OXA-51, TEM	ST208
72	2015	Vienna/ North Austria	Catheter tip	MDR	I	R	R	R	R	R	R	R	R	R	S	S	pos	OXA-23-like, OXA-58-like, OXA-51, TEM	ST195
73	2015	Vienna/ North Austria	Bronchoalveolar lavage	MDR	I	R	R	R	R	R	R	R	R	R	S	S	pos	OXA-23-like, OXA-58-like, OXA-51, TEM	ST195
74	2015	Vienna/ North Austria	Rectal swab	MDR	R	R	R	R	R	R	R	R	S	R	S	S	neg	OXA-23-like, OXA-58-like, OXA-51, TEM	ST195
75	2015	Vienna/ North Austria	Wound swab	MDR	S	R	R	R	R	R	R	R	R	R	S	S	pos	OXA-23-like, OXA-58-like, OXA-51, TEM	ST502
76	2015	Vienna/ North Austria	Bronchoalveolar lavage	MDR	R	R	R	R	R	R	S	R	R	R	S	S	pos	OXA-51	ST350

77	2015	Vienna/ North Austria	Bronchoalveolar lavage	MDR	I	R	R	R	R	R	R	R	R	R	S	S	pos	OXA-23-like, OXA-51	ST195
78	2015	Vienna/ North Austria	Tracheal aspirate	XDR	R	R	R	R	R	R	R	R	R	R	S	R	pos	OXA-23-like, OXA-51	ST2026
79	2015	Vienna/ North Austria	Tracheal aspirate	MDR	R	R	R	R	R	R	R	R	R	R	S	S	pos	OXA-23-like, OXA-51	ST195
80	2016	Vienna/ North Austria	Wound swab	XDR	R	R	R	R	R	R	R	R	R	R	R	S	pos	OXA-23-like, OXA-58-like, OXA-51	ST208
81	2016	Vienna/ North Austria	Decubital ulcer	MDR	R	R	R	R	R	R	R	R	R	R	S	S	pos	OXA-51	ST208
82	2016	Vienna/ North Austria	Wound swab	MDR	S	R	R	R	R	R	R	R	R	R	S	S	neg	OXA-23-like, OXA-51	ST195
83	2016	Vienna/ North Austria	Bronchoalveolar lavage	MDR	R	R	R	R	S	R	R	R	R	R	S	S	neg	OXA-58-like, OXA-51, TEM	ST208
84	2016	Vienna/ North Austria	Throat swab	XDR	I	R	R	R	R	R	S	R	S	R	S	R	pos	OXA-51	ST231
85	2016	Vienna/ North Austria	Wound swab	XDR	R	R	R	R	R	R	R	R	R	R	S	R	pos	OXA-58-like, OXA-51, TEM	ST195
86	2016	Vienna/ North Austria	Axilla	MDR	R	R	R	R	R	R	R	R	R	R	S	S	pos	OXA-23-lie, OXA-58-like, OXA-51, TEM	ST231
87	2016	Vienna/ North Austria	Catheter tip	MDR	R	R	R	R	R	R	R	R	R	R	S	S	neg	OXA-58-like, OXA-51, TEM	ST218
88	2016	Vienna/ North Austria	Bronchoalveolar lavage	MDR	R	R	R	R	R	R	R	R	R	R	S	S	neg	OXA-51	ST218
89	2016	Vienna/ North Austria	Rectal swab	MDR	R	R	R	R	R	R	R	R	R	R	S	S	neg	OXA-51, TEM	ST231
90	2016	Vienna/	Wound swab	XDR	S	R	R	R	R	R	R	R	R	R	S	R	pos	OXA-51	ST231

		North Austria																	
91	2016	Vienna/ North Austria	Groin	XDR	R	R	R	R	R	R	R	R	R	R	S	R	neg	OXA-58-like, OXA-51	ST195
92	2016	Vienna/ North Austria	Tracheal aspirate	MDR	R	R	R	R	R	R	R	R	R	R	S	S	pos	OXA-58-like, OXA-51, TEM	ST209
93	2017	Vienna/ North Austria	Decubital ulcer	MDR	S	R	R	R	R	S	S	S	S	R	S	S	neg	OXA-51	ST447
94	2017	Vienna/ North Austria	Tracheal aspirate	MDR	R	R	R	R	R	R	R	R	R	R	S	S	pos	OXA-51, TEM	ST425
95	2017	Vienna/ North Austria	Urine	MDR	S	R	R	R	R	S	S	S	R	R	S	S	neg	OXA-51	ST425
96	2017	Vienna/ North Austria	Rectal swab	MDR	R	R	R	R	R	R	R	R	R	R	S	S	pos	OXA-51, TEM	ST425
97	2017	Vienna/ North Austria	Wound swab	MDR	R	R	R	R	R	R	R	R	R	R	S	S	pos	OXA-51	ST502
98	2017	Vienna/ North Austria	Catheter tip	XDR	R	R	R	R	R	R	R	R	R	R	R	S	pos	OXA-51, TEM	ST425
99	2017	Vienna/ North Austria	Wound swab	MDR	R	R	R	R	R	R	R	R	R	R	S	S	pos	OXA-51, TEM	ST425
100	2017	Vienna/ North Austria	Tracheal aspirate	MDR	R	R	R	R	R	R	R	R	R	R	S	S	pos	OXA-51, TEM	ST425
101	2017	Vienna/ North Austria	Throat swab	MDR	S	R	R	R	R	R	S	S	R	R	S	S	neg	OXA-51	ST208
102	2017	Vienna/ North Austria	Bronchoalveolar lavage	MDR	R	R	R	R	R	R	R	R	R	R	S	S	pos	OXA-51, TEM	ST812
103	2017	Vienna/	Bronchoalveolar	MDR	S	R	R	R	S	R	S	S	S	S	S	S	neg	OXA-51	ST930

		North Austria	lavage																
104	2017	Vienna/ North Austria	Wound swab	MDR	R	R	R	R	R	R	R	R	R	R	S	S	pos	OXA-51	ST231
105	2017	Vienna/ North Austria	Rectal swab	MDR	I	R	R	R	R	R	R	R	R	R	S	S	pos	OXA-51	ST502
106	2017	Vienna/ North Austria	Bronchoalveolar lavage	MDR	S	R	R	R	R	R	R	R	R	R	S	S	pos	OXA-51	ST812
107	2017	Vienna/ North Austria	Wound swab	MDR	I	R	R	R	R	R	R	R	R	R	S	S	pos	OXA-51	ST502
108	2017	Vienna/ North Austria	Wound swab	MDR	R	R	R	R	R	R	R	R	R	R	S	S	pos	OXA-51	ST281
109	2017	Vienna/ North Austria	Bronchoalveolar lavage	MDR	R	R	R	R	R	R	R	R	R	R	S	S	pos	OXA-51	ST502
110	2017	Vienna/ North Austria	Groin	MDR	R	R	R	R	R	R	R	R	R	R	S	S	pos	OXA-51	ST195
111	2017	Vienna/ North Austria	Rectal swab	MDR	I	R	R	R	R	R	R	R	R	R	S	S	pos	OXA-51	ST502
112	2017	Vienna/ North Austria	Bloodculture	MDR	R	R	R	R	R	R	R	R	R	R	S	S	neg	OXA-23-like, OXA-51	ST195
113	2017	Vienna/ North Austria	Decubital ulcer	XDR	I	R	R	R	R	R	R	R	R	R	R	S	pos	OXA-24-like, OXA-51	ST231
114	2017	Vienna/ North Austria	Bloodculture	MDR	R	R	R	R	R	R	R	R	R	R	S	S	pos	OXA-23-like, OXA-51, TEM	ST195
115	2015	Vienna/ North Austria	Bronchoalveolar lavage	MDR	S	R	R	R	S	I	S	S	S	I	S	S		OXA-143	*A. pittii*
116	2015	Vienna/	Wound swab	MDR	S	R	R	R	S	I	S	S	S	I	S	S		OXA-23-like, TEM	*A. pittii*

117	2017	North Austria Vienna/ North Austria	Bloodculture	MDR	S	R	R	R	R	R	S	S	S	I	S	S	-	*A. pittii*

Carbapenem resistant Acinetobacter spp in Austria, *A. baumannii* (No 1-114), *A. pittii* (No 115-117), MDR-multi-drug resistant; XDR- extensively drug resistant; PDR-pandrug resistant. SAM- Ampicillin/Sulbactam; PT- Piperacillin/Tazobactam, CAZ-Ceftazdime; FEP- Cefepime; IMP- Imipenem; MEM-Meropenem; GM- Gentamicin; AN- Amikacin; SXT- Trimethoprim/Sulfamethoxazole; CIP- Ciprofloxacin; CL- Colistin; TGC- Tigecycline.
